# The soluble loop BC region guides, but not dictates, the assembly of the transmembrane cytochrome b_6_

**DOI:** 10.1371/journal.pone.0189532

**Published:** 2017-12-14

**Authors:** Lydia Tome-Stangl, Cornelia Schaetzel, Stefan Tenzer, Frank Bernhard, Dirk Schneider

**Affiliations:** 1 Institute of Pharmacy and Biochemistry, Johannes Gutenberg-University Mainz, Mainz, Germany; 2 Institute of Immunology, University Medical Center Mainz, Mainz, Germany; 3 Institute of Biophysical Chemistry, Goethe University Frankfurt am Main, Frankfurt am Main, Germany; Centre National de la Recherche Scientifique, Aix-Marseille Université, FRANCE

## Abstract

Studying folding and assembly of naturally occurring α-helical transmembrane proteins can inspire the design of membrane proteins with defined functions. Thus far, most studies have focused on the role of membrane-integrated protein regions. However, to fully understand folding pathways and stabilization of α–helical membrane proteins, it is vital to also include the role of soluble loops. We have analyzed the impact of interhelical loops on folding, assembly and stability of the heme-containing four-helix bundle transmembrane protein cytochrome b_6_ that is involved in charge transfer across biomembranes. Cytochrome b_6_ consists of two transmembrane helical hairpins that sandwich two heme molecules. Our analyses strongly suggest that the loop connecting the helical hairpins is not crucial for positioning the two protein “halves” for proper folding and assembly of the holo-protein. Furthermore, proteolytic removal of any of the remaining two loops, which connect the two transmembrane helices of a hairpin structure, appears to also not crucially effect folding and assembly. Overall, the transmembrane four-helix bundle appears to be mainly stabilized via interhelical interactions in the transmembrane regions, while the soluble loop regions guide assembly and stabilize the holo-protein. The results of this study might steer future strategies aiming at designing heme-binding four-helix bundle structures, involved in transmembrane charge transfer reactions.

## Introduction

Almost three decades ago, J.-L. Popot and D.M. Engelman proposed a simple model, describing the folding pathway of membrane proteins [1, 2], and this model has served many times as a valuable concept guiding the design of artificial transmembrane (TM) proteins. Based on the two-stage model, stable TM α-helices initially integrate independently into a membrane (stage one), and subsequently sequence specific interactions between TM helices result in formation of a fully and functionally folded protein (stage two). However, in recent years multiple structures of α-helical membrane proteins have been resolved, and based on these structures it became evident that folding of α-helical membrane proteins cannot be described by a simple two-step process in many cases [3–5]. Nevertheless, the two-stage model has proven to properly describe folding and assembly of several proteins with “simple” helix bundle structures [2, 6]. In cases where folding of these proteins involves cofactor binding, insertion of peripheral domains or quaternary structure formation, the original two-stage model was extended to cover these additional folding steps [7–10].

When a polypeptide chain contains multiple TM helices, extra-membranous loops covalently connect the individual TM helices, and the loops´ length and flexibility might define the helices´ translational and rotational freedom. Thus, especially short loops can be necessary to bring helices in close proximity, thereby promoting TM helix packing. While the number of amino acids in a loop can vary from just a few up to over one hundred [11, 12], a longer loop does not inevitably lead to an increased interhelical distance compared to a shorter loop, and the distance between the linked TM helices does not automatically correlate to the length of the loop [13]. Instead, the exact distance between two TM helices appears to be an exclusive question of helix packing [13]. However, for some α-helical membrane proteins it has been shown that loops can be important for assembly and stability of the proteins [14]. Hence, modifications of a loop, such as alteration of the amino acid sequence, variation of the loop´s length or cleavage of the loop, might not only seriously influence the stability, but also folding and function of a membrane protein.

Loops can promote the assembly of individual TM helices and in some cases can even be vital for mediating and stabilizing helix-helix interactions. For bacteriorhodopsin it has *e*.*g*. been shown that none of its individual loops are essential for protein assembly [15–19]. Bacteriorhodopsin can assemble from protein fragments, although the assembled protein is less stable when compared to the wild type (wt) protein [17, 20]. In fact, molecular dynamic simulations of a helix-loop-helix motif from bacteriorhodopsin have indicated that loop deletion only remotely leads to structural and dynamic changes in the helices [21]. However, this effect is even limited to the termini of the helix, and interfacial regions are able to counterbalance the loss of inter-helical interactions caused by loop removal. Similarly, the cytoplasmic loops of rhodopsin are not vital for protein assembly [22]. In contrast, cleavage of the extracellular loops of the retinal visual pigment of mammals prevented protein assembly, indicating that the role of loops can severely differ [23, 24]. The deleterious effect observed upon cleavage of an extracellular loop could be caused by an increased helical flexibility and movement and, as a consequence, decreased intramolecular interactions.

In the present study, we have explored the impact of interhelical loops on folding, assembly and stability of the cofactor-containing TM protein cytochrome b_6_ (cyt. b_6_). Cyt. b_6_ is a core subunit of the cyt. b_6_f complex that is involved in charge transfer across thylakoid membranes. The protein consists of four TM helices, which are covalently connected via three loops [25–27]. Two non-covalently bound heme cofactors are localized in the heme-binding pocket between two cyt. b_6_ “halves” formed by the helical hairpins AB and CD, and thus the four-helix bundle structure is stabilized by the heme molecules. Previous studies with TM b-type cytochromes, such as cyt. b_6_ [8, 28–32] or cyt. b_559_ [9, 33–37] have indicated that the respective holo-cytochromes spontaneously assemble *in vitro* and *in vivo* in presence of heme. The two b-type hemes of *in vitro* reconstituted cyt. b_6_ have redox potentials of -187 mV (the low-potential heme b_L_) and -68 mV (the high-potential heme b_H_), respectively [8], which is in perfect agreement with the potentials determined for cyt. b_6_ isolated from spinach thylakoid membranes [38] and cyt. b_6_ embedded within the *Escherichia coli* (*E*. *coli*) inner membrane [39]. Thus, cyt. b_6_ can be well reconstituted and studied *in vitro*. In fact, cyt. b_6_ and the homologous four N-terminal TM helices of cyt. b have served many times as blueprints for the *de novo* design of soluble as well as of TM b-type cytochrome-like structures (e.g. [40–47].

In the past years, many aspects of the cyt. b_6_ folding pathway have been described [8, 29, 48, 49]. The TM apo-cyt. b_6_ inserts into a membrane independent of the cofactor, and after addition of heme to isolated membranes, spontaneous assembly of holo-cyt. b_6_ was observed [29]. These observations imply that the membrane-integrated apo-protein adopts a conformation that allows efficient heme incorporation. Subsequently, stepwise binding of the two non-covalently bound heme molecules was shown both, *in vivo* and *in vitro* [8, 50, 51]. Thus, the BC loop connecting TM helices AB and CD, respectively, might be crucial for formation of an open, clamshell-like structure, competent for heme binding. Formation of the holo-cytochrome is mediated by the heme cofactors and not only by TM helix-helix interactions, and it is therefore likely that the loop connecting the two helical hairpins has a more important role for directing helix interactions and cofactor binding. But how important are the loops really for assembly of the TM b-type cytochrome?

In our analyses, we mainly focused on the loop connecting the helical hairpins AB and CD. The here presented results show that neither elongation nor elimination of the loop connecting the two hairpins (halves) of cyt. b_6_ (AB and CD) has an impact on folding and assembly of the holo-protein. In fact, a cyt. b_6_-like protein can be assembled from two fragments AB and CD and heme. Thus, the BC loop appears to not be crucial for positioning or orienting the halves during the folding pathway. Furthermore, removing any of the remaining two loops by protease cleavage appears also not to affect the stability of cyt. b_6_. Likely, the holo-cytochrome can assemble solely from the TM regions and free heme. Thus, the soluble loop regions solely guide and stabilize the assembly of the heme-binding TM four-helix bundle structure.

## Material and methods

### Site-directed mutagenesis

Construction of the plasmid pRHMb_6_, coding for the *Spinacia oleracea L* cyt. b_6_ protein, N-terminally fused to the maltose-binding protein (MBP) of *E*. *coli*, has been described previously [30]. Mutation of the cyt. b_6_ amino acids His 86 and His 187 to alanine is described in [8]. For insertion of multiple Gly residues in the BC-loop, an overlap extension polymerase chain reaction (PCR) was performed. In two separate PCRs, the gene was mutated in two partly complementary parts. The resulting PCR products overlap in the area of the newly inserted mutation. The primers contained codons for Gly at the 5´-ending and a 3´-ending complementary to the cyt. b_6_ coding sequence before or after the point of insertion (between Pro 113 and Arg 114) (S1 Table, No. 1–4). In a third PCR, the 3´-ending of one of the products from the first PCRs served as primer for synthesis of the remaining PCR product and *vice versa*, and thus as a result the two parts of the mutated gene were connected. For unmodified endings of this third fragment, primers were used that were complementary to regions coding for the N- or C-terminus of cyt. b_6_, respectively (S1 Table, No. 5–6). After restriction digestion by *Sac*I and *Bam*HI, the product of the third PCR was ligated into the equally restriction digested vector pRHMb_6_, resulting in the plasmids pRHMb_6_G_5_ and pRHMb_6_G_10_.

### Expression, purification and reconstitution of cyt. b_6_

Heterologous expression of cyt. b_6_ in *E*. *coli* HMS174(DE3) and subsequent purification from inclusion bodies, folding and reconstitution with heme for holo-protein formation were performed as described [30]. The proteins carrying mutations of His 86 and His 187 [8] or elongated BC-loops were purified as the wt protein. If needed, purified cyt. b_6_ was mixed with heme in excess and a subsequent purification step was performed via immobilized metal affinity chromatography (IMAC) to remove unbound heme. The purification was essentially performed as described [30], and the Ni- nitrilotriacetic acid (NTA)-matrix (Qiagen) was equilibrated with 50 mM Tris pH 8.0, 50 mM NaCl and 1 mM *n*-dodecyl β-D-maltoside (DDM). Protein concentrations were determined via the bicinchoninic acid (BCA) protein assay [52] with a Pierce BCA Protein Assay Kit from Thermo Scientific (Rockford, USA) and BSA as standard protein for calibration.

In addition, cyt. b_6_ and the two halves, cyt. b_6_-AB and cyt. b_6_-CD, were obtained by cell-free synthesis in a continuous exchange cell-free system (CECF) without the MBP fusion tag [53]. The proteins were synthesized in the precipitate forming mode without addition of detergents or lipids. The precipitated proteins were harvested after incubation by centrifugation at 10,000 g for 10 min at 4°C and solubilized in 10 mM Tris, 100 mM sodium phosphate buffer pH 8.0 and 50 mM sodium dodecyl sulfate (SDS). IMAC-purification with Ni-NTA, folding and reconstitution with heme were performed as described for the cyt. b_6_ fusion protein. Heme solutions were prepared as described in [30] and concentrations were determined spectroscopically at 385 nm utilizing the extinction coefficient of 56 mM^-1^ cm^-1^ [54].

Heme-binding ratios were determined via titration experiments, and the stability of the reconstituted proteins was analyzed at increasing SDS concentrations, as described in [8, 30]. After each step, absorbance spectra were recorded under air-oxidized conditions and after reduction with 5 mM sodium dithionite. The relative absorbance changes were calculated as the ratio of the absorbance at 414 nm to 404 nm, representing the quotient of the maxima of the Soret-band of bound to unbound heme.

### Proteolytic digestion of cyt. b_6_

Both, wt cyt. b_6_ and apo-cyt. b_6_ H86A H187A, were proteolyzed at room temperature using 0.02 mg proteinase K (Serva, Heidelberg) per mg protein. Aliquots were taken for analysis at distinct time points, and the digestion was stopped with 5 mM phenylmethanesulfonyl fluoride. Absorbance spectra were recorded from 350 to 700 nm with a band width of 0.5 nm directly, in the case of reconstituted holo-protein, or after addition of 5 μM heme, in case of the apo-protein, under air-oxidized conditions and after reduction with 5 mM sodium dithionite. The holo-cyt. b_6_ fragments remaining after 5 h of proteolysis were separated by gel filtration, using a Superose™ 12 10/300 GL column (GE Healthcare) with buffer containing 50 mM Tris pH 8.0, 50 mM NaCl and 1 mM DDM, and were analyzed by mass spectrometry.

### Ultra-performance liquid chromatography

Capillary liquid chromatography of tryptic peptides was performed with a Waters NanoAcquity Ultra-Performance Liquid Chromatography system equipped with a 200 μm x 5 mm PepSwift Monolithic Column (PS-DVB) thermostated at 55°C and a 2.6 μL PEEKSIL-sample loop (SGE, Darmstadt, Germany). The aqueous mobile phase (mobile phase A) was H_2_O (LC-MS Grade, Roth, Freiburg, Germany) with 0.1% formic acid. The organic mobile phase (mobile phase B) was 0.1% formic acid in acetonitrile (LC-MS grade, Roth). Sample (1 μL injection) were loaded onto the column in direct injection mode with 1% mobile phase B for 4 min at 800 nL/min. Peptides were eluted from the column with a gradient from 1–50% mobile phase B over 40 min at 800 nL/min followed by a 7 min rinse of 99% mobile phase B. The column was immediately re-equilibrated at initial conditions (1% mobile phase B) for 8 min. [Glu^1^]fibrinopeptide was used as lockmass at 100 fmol/μL. Lockmass solution was delivered from the auxiliary pump of the NanoAcquity system at 500 nL/min to the reference sprayer of the NanoLockSpray source.

### Mass spectrometry analysis

Mass spectrometric analysis of tryptic peptides was performed using a Synapt G2-S mass spectrometer (Waters Corporation) operated in v-mode with a typical resolution of at least 25,000 full width half maximum in positive mode electrospray ionization. The time of flight analyzer of the mass spectrometer was externally calibrated with a sodium iodide mixture from *m/z* 50 to 1990. The data were post-acquisition lock mass corrected using the doubly charged monoisotopic ions of [Glu^1^]-fibrinopeptide B. Accurate mass liquid chromatography–mass spectrometry data were collected in data-independent mode of acquisition in combination with on-line ion mobility separations as described before [55].

### Data processing and peptide identification

Continuum LC-MS data were processed and searched using ProteinLynx GlobalSERVER version 3.0.2 (Waters Corporation). Peptide identifications were obtained by searching a custom-compiled database containing cytochrome sequences and *E*. *coli* proteins. Sequence information of proteinase K, and human keratins were added to the databases. The experimental data were typically searched with a three ppm precursor and ten ppm product ion tolerance, using settings for unspecific proteolytic digestion and variable methionine oxidation set as modification. Peptides with a minimum PLGS (ProteinLynx Global server) identification score of 6.5 (corresponding to a False Discovery Rate FDR <0.1% on peptide level) were used for further analysis.

### SDS-PAGE and Western blot analyses

SDS- polyacrylamide gel electrophoresis (PAGE) was used to separate protein samples according to their electrophoretic mobility [56]. The separated proteins were stained with Coomassie Brilliant Blue R250 or electro-blotted on a polyvinylidene difluoride membrane (Roth) with a semi-dry blotter (Bio-Rad) [57]. For Western blot analyses, antibodies directed against the His-tag (monoclonal, horseradish peroxidase (HRP)-conjugated, Novagen, 1:1000), the cyt. b_6_ N-terminus (SKVYDWFEER, polyclonal, rabbit, Gramsch Laboratories, Munich, Germany, 1:10000) or the cyt. b_6_ C-terminus (EIRKQGISGPL, polyclonal, rabbit, Gramsch Laboratories, Munich, Germany, 1:10000) were used. In case of the latter ones, anti-rabbit antiserum (HRP-conjugated (1:10000), Sigma-Aldrich) was used as a secondary antibody. If HRP-conjugated antibodies were used, membranes were developed using the Pierce ECL Western Blotting Substrate (Thermo Fisher Scientific Inc.), and chemiluminescence was detected using a Stella 3200 (Raytest).

## Results

### BC-loop prolongation does not affect the assembly and stability of cyt. b_6_

**Fig 1:**
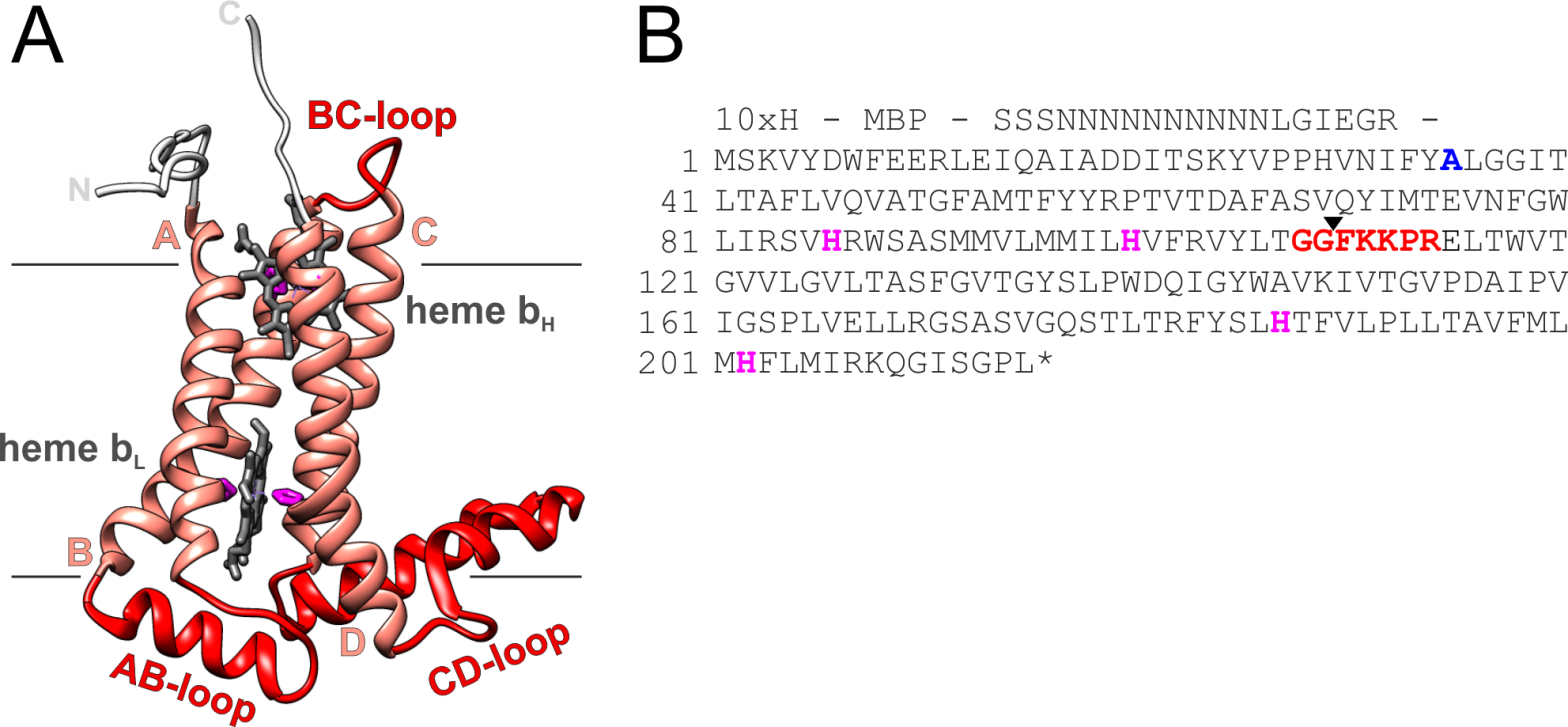
**Structure (A) and amino acid sequence (B) of cyt. b**_**6**_. (A) Cyt. b_6_ consists of four TM helices (A to D, in rose) that bind two heme molecules non-covalently (heme b_H_ and heme b_L_, gray) via histidine residues (pink) located in helices B and D. Separated by the cofactors, cyt. b_6_ is composed of two halves, consisting of the helices A and B and the helices C and D, respectively. The ribbon presentation is oriented as viewed from the plane of the (thylakoid) membrane and with the side of the chloroplast stroma on top. Both, C- and N-terminus are facing the stroma. The interhelical loops (red) are located within the stroma (BC loop) as well as within the thylakoid lumen (AB loop and CD loop) (PDB-ID 1VF5 from [27]). (B) Amino acid sequence of cyt. b_6_ from *Spinacia oleracea L* (underlined) with a deca-histidine-tag and the maltose-binding protein (MBP) of *E*. *coli* fused to the N-terminus (indicated). Amino acids discussed in the text are highlighted in bold. Blue: Ala 35. Originally, there is a Cys at this position, which covalently binds a third heme (heme c_n_) *in vivo*. Hovewer, heme c_n_ is not essential for the assembly of the b-type cytochrome [8]. Since we analyzed assembly of cyt. b_6_ by measuring the absorbance of the two non-covalently bound hemes b_L_ and b_H_, we analyzed the C35A mutant and refer to it as “wt”. Pink: His 86, His 100, His 187 and His 202. These residues serve as axial ligands for the two non-covalent bound hemes. Red: Gly 108 to Arg 114. These amino acids form the BC-loop. To unravel the role of this loop, the BC-loop was elongated by five or ten Gly residues between Pro 113 and Arg 114. The black arrow between Gly 109 and Phe 110 highlights the boundaries of cf-cyt. b_6_-AB and cf-cyt. b_6_-CD.

Cyt. b_6_ has four TM α-helices that are connected by loops, and two heme molecules are non-covalently bound via four histidine residues (Fig 1A) [25, 58]. *In vivo*, a third heme, heme c_n_, is covalently bound via a cysteine residue to the periphery of cyt. b_6_ (Cys 35 in spinach cyt. b_6_) [25, 58]. However, we have previously shown that heme c_n_ is non-essential for holo-protein assembly [8], and a mutation of Cys 35 to alanine does not affect the amount of heme bound to the purified and reconstituted holo-protein [8, 30]. Thus, to completely eliminate the possibility that a third heme would eventually bind in our experiments, we used the cyt. b_6_ C35A mutant in our experiments and in the following we refer to this protein as “wt” (Fig 1B). The cyt. b_6_ TM helices A and B as well as the helices C and D form coiled coil structures in the holo-protein structure, and throughout this paper, we will refer to these helical hairpins as cyt. b_6_ “halves”. The two non-covalently bound hemes are sandwiched in between these two halves.

**Fig 2:**
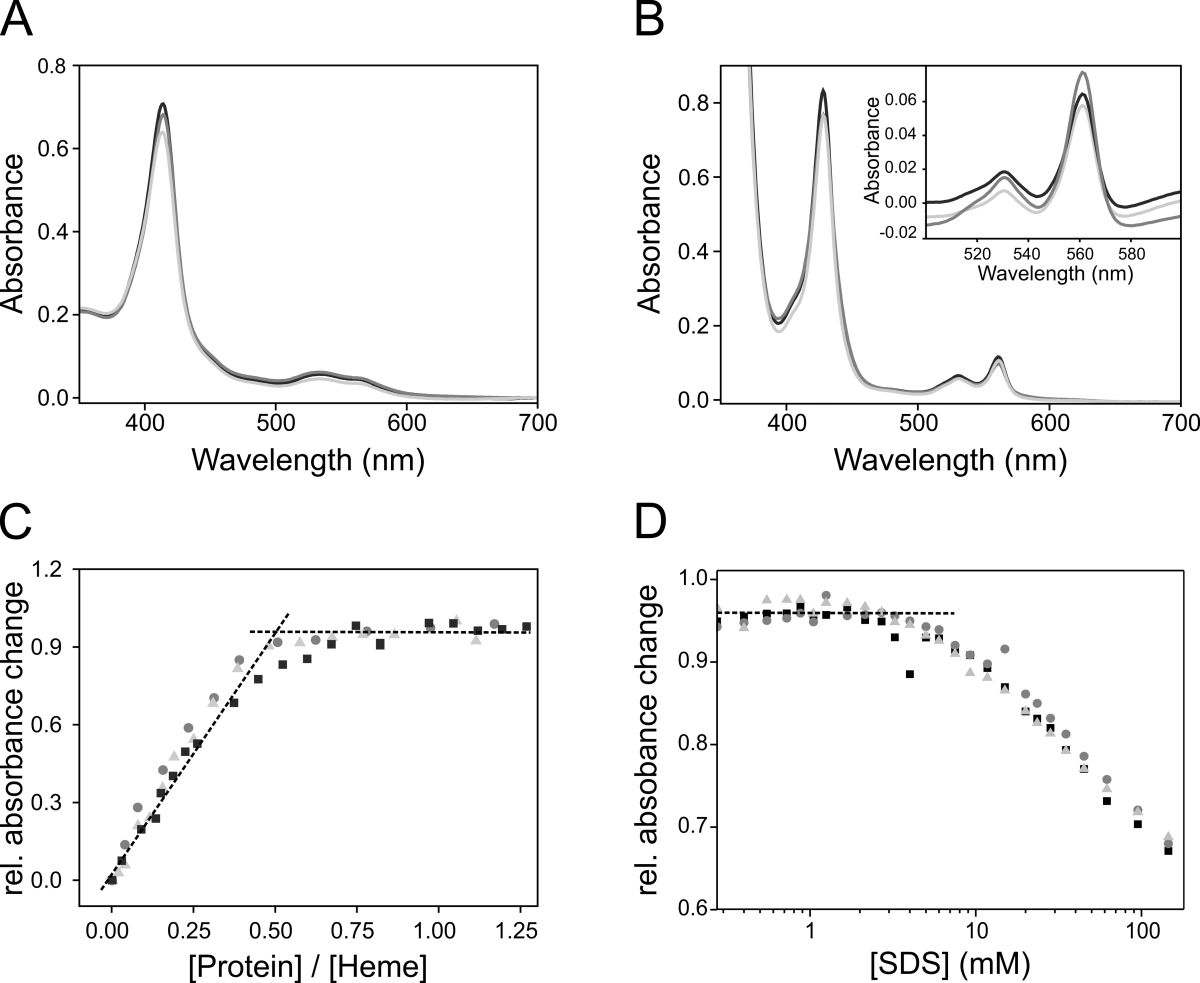
*In vitro* reconstitution of cyt. b_6_ and its G_n_-mutants containing prolonged BC loops. Absorbance spectra of *in vitro* reconstituted cyt. b_6_ (black), cyt. b_6_-G_5_ (dark gray) and cyt. b_6_-G_10_ (light gray) under oxidizing (A) and reducing (B) conditions. G_5_ and G_10_ stands for the number of Gly residues inserted into the BC-loop. The absorbance maxima are essentially identical for all three proteins (A: 561 nm / 532 nm / 414 m; B: 562 nm / 532 nm / 429 nm for the α-/β-/γ-band). The same applies for the redox difference spectra (562 nm / 532 nm for the α-/β-band) (inlet B). (C) and (D) show titrations of cyt. b_6_ (black squares), cyt. b_6_-G_5_ (dark gray spheres) and cyt. b_6_-G_10_ (light gray triangles) with heme (C) or SDS (D) under oxidizing conditions. Each data point represents an individual measurement. The quotient of the absorbance intensity at 414 nm to 404 nm was calculated, representing the absorbance maximum of the Soret-band of bound and unbound heme, respectively, and normalized to “0” for solely free heme and “1” for solely bound heme. (C) Increasing amounts of protein were titrated into buffer containing 5 μM heme to measure heme-binding isotherms. For all three proteins, the titration experiments show a saturation at a protein/heme ratio of 0.5 and higher. (D) To determine the proteins´ stability, increasing amounts of SDS were added to reconstituted protein. All three cyt. b_6_ variants are stable up to an SDS concentration of ~4.5 mM.

To unravel the role of individual loops connecting the cyt. b_6_ TM helices, we first evaluated the impact of the loop connecting the two cyt. b_6_ halves (AB and CD) on folding and stability of cyt. b_6_, and we asked whether extending this loop has an impact on holo-protein assembly. As can be seen in the cyt. b_6_ crystal structure, the BC-loop is relatively small, consisting of solely seven amino acids with the sequence ^108^Glu-Glu-Phe-Lys-Lys-Pro-Arg^114^ [25, 27]. Because of this, we anticipated that the loop might be crucial for keeping the two cyt. b_6_ halves in close proximity prior to heme binding. With the help of overlap extension PCR, the BC-loop was elongated by five or ten Gly residues between Pro 113 and Arg 114. Thereby the length of the BC-loop was more than doubled. The prolongation increased the flexibility of the loop stepwise and thereby allowed an increased spatial distance between the two halves of the TM cytochrome. Cyt. b_6_, cyt. b_6_-G_5_ and cyt. b_6_-G_10_ were heterologously expressed in *E*. *coli* as MBP-fused proteins and purified for further analyses (S1 Fig). It is well established that the formation of b-type holo-cytochromes can be properly visualized spectroscopically (e.g. [9, 29, 30, 34, 41, 42, 59–62]). The Soret- or γ-band at 400 to 430 nm is the most dominant band in the absorbance spectrum of free heme and of heme-containing proteins. If heme is properly incorporated in b-type cytochromes, the coordination sphere of heme changes due to heme binding to a protein via His or Met residues. This leads to a low-spin, six-fold-coordinated iron in the center of the heme porphyrin ring [63], which becomes evident as a red shift in the Soret-band absorbance maximum compared to free, unbound heme under both, oxidizing and reducing conditions. Furthermore, especially under reducing conditions the Soret-band of b-type cytochromes is sharpened compared to free, unbound heme and the splitting of the α/β band region at 500 to 600 nm is far more pronounced. All these features are together diagnostic for proper *in vivo* and *in vitro* formation of b-type holo-cytochromes. In Fig 2A and 2B, absorbance spectra of *in vitro* reconstituted apo-cyt. b_6_ and its G-mutants are shown under oxidizing and reducing conditions. All three proteins have the γ-, β- and α-absorbance maxima, characteristic for b-type cytochromes, at identical wavelengths. Under oxidizing conditions, the bandwidth of the Soret band at half maximum (*fwhm*) increased slightly for cyt. b_6_ with an elongated BC-loop (cyt. b_6_ wt: 24.0 nm, cyt. b_6_-G_5_:27.4 nm, cyt. b_6_-G_10_: 27.1 nm) indicating that the heme environment is slightly less well defined in the G-mutants under oxidizing conditions compared to the wt protein. However, under reducing conditions, the *fwhm* remained essentially constant with 22.6 nm for the wt cyt. b_6_ and 22.5 nm and 22.1 nm for the cyt. b_6_-G_5_ and cyt. b_6_-G_10_ mutants. Also, the redox difference spectra show identical peak maxima for all three proteins (inlet in Fig 2B), indicating that the heme environments are essentially identical. The peak maxima and *fwhm*-values are in good agreement with previously described values obtained after analyses of purified cyt. b_6_ in DDM-micelles [8, 29–32, 39], membrane embedded cyt. b_6_ [29] as well as cyt b_6_ isolated from the cytochrome b_6_f complex from spinach chloroplasts [64, 65] or cyanobacteria [66]. Thus, the spectral properties indicated stable and proper assembly of all reconstituted cyt. b_6_ G-mutants. However, the slight absorbance decrease of the γ-band and the small increase in *fwhm*-values of the oxidized cyt. b_6_ G-mutants (Fig 2A and 2B) might indicate slightly less rigid binding of the hemes with increasing length of the BC-loop.

To further reveal potential differences in heme binding and assembly of the G-mutants, the protein-to-heme-binding ratios were determined for all three proteins. Therefore, isotherms for heme binding to apo-cyt. b_6_ were determined by titrating a solution of heme with increasing amounts of protein (Fig 2C). The relative absorbance intensity increased for all three cyt. b_6_ variants with increasing protein concentrations until a plateau was reached, and further addition of protein resulted in stable relative absorbance levels. From this point on, all available heme molecules were bound. Cyt. b_6_, cyt. b_6_-G_5_ and cyt. b_6_-G_10_ have an essentially identical heme-binding behavior, and a protein-to-heme-ratio of ~0.5 was determined for all proteins, meaning that two heme molecules are bound by one protein molecule. This confirms the observation that the heme-binding affinity of cyt. b_6_ is essentially not affected by the BC-loop prolongation.

Next, the stability of the three cyt. b_6_ variants was assessed by stepwise denaturation of the TM protein by addition of increasing amounts of SDS, an established method, allowing to estimate the thermodynamic stability of α-helical TM proteins [67–69]. Addition of SDS to a TM protein solubilized in a non-ionic detergent, here DDM, results in separation of the TM α-helices, which causes loss of the tertiary contacts, whereas the α-helical secondary structure typically remains intact. This SDS-induced denaturation in principle is a reverse of stage two of the two-stage model for protein folding. In case of cyt. b_6_ and its G-mutants, separation of the TM helices leads to a release of the heme cofactors, which can be detected by a decreased Soret-band absorption intensity (Fig 2D) [30]. At low SDS concentrations of up to 4.5 mM, no heme is released and the titration curve shows a plateau. Only at concentrations >4.5 mM SDS, the relative absorbance steadily decreases, indicating that the helices start to dissociate and release heme. Importantly, this commences at the same SDS concentration for all three proteins, which again indicates that elongation of the BC-loop does no effect the stability and heme-binding capacity of cyt. b_6_.

Together, cyt. b_6_-G_5_ and cyt. b_6_-G_10_ have spectral properties like the wt protein. Furthermore, the protein-to-heme-binding ratio and the proteins´ stability against SDS-induced unfolding are also identical to the wt cyt. b_6_ protein. Thus, our data indicate that an extended BC-loop does essentially not affect the stability and heme-binding capacity of cyt. b_6_, despite an increased distance and flexibility of the two cyt. b_6_ halves. Hence, a special proximity of the two helical hairpins AB and CD, mediated by the BC-loop, appears to not be essential for assembly and stabilization of cyt. b_6_.

### Cyt. b_6_ halves assemble to form the holo-protein

**Fig 3:**
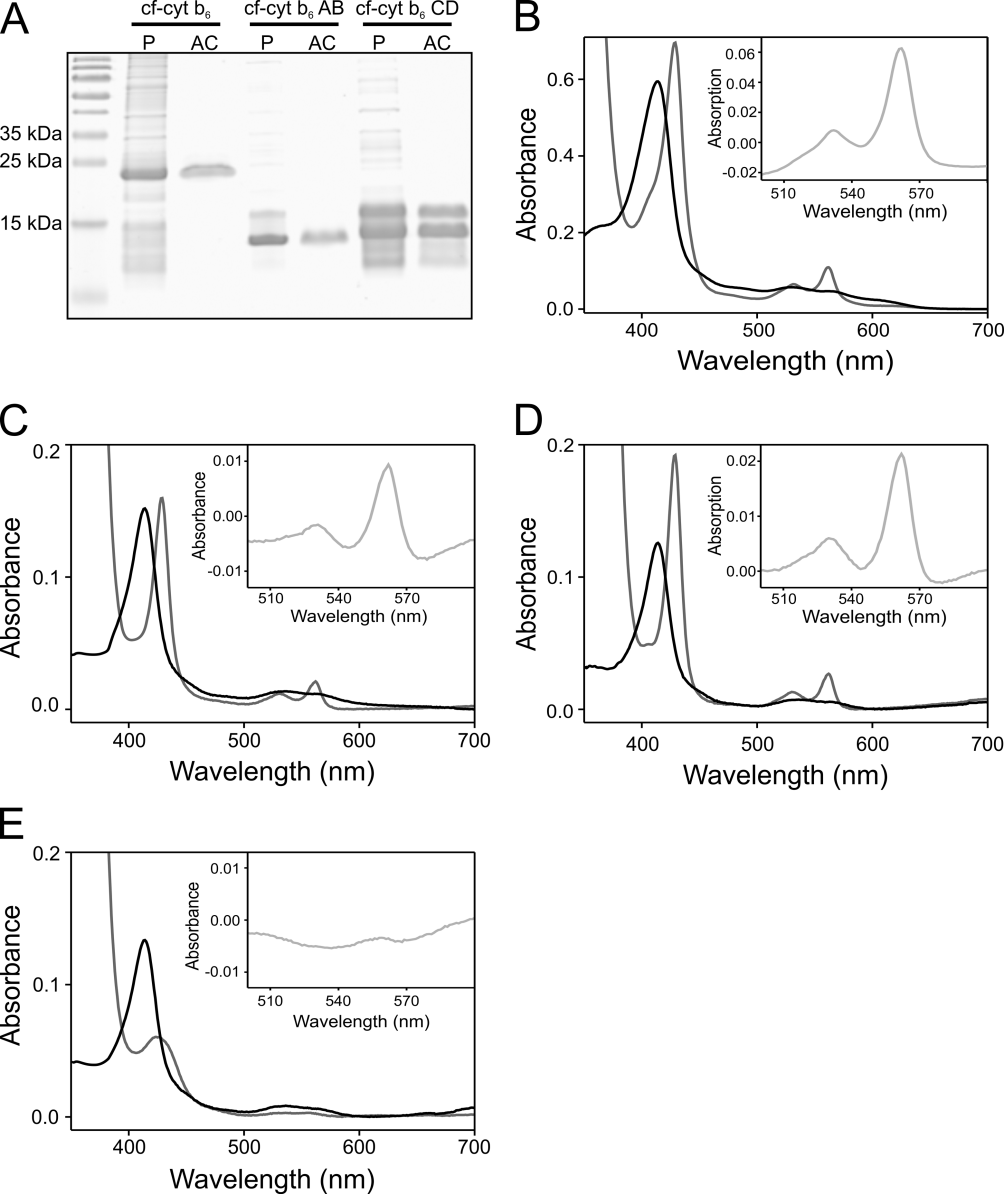
Purification of cf-cyt. b_6_ and the two cyt. b_6_ halves cf-cyt. b_6_-AB and cf-cyt. b_6_-CD, and assembly of the cyt. b_6_ holo-protein. (A) Proteins were separated on an 18% SDS gel. M: molecular mass standard; P: cell-free expressed protein; AC: protein purified by affinity chromatography. (B to E) UV/VIS absorbance spectra were acquired under oxidizing (black) and reducing (gray) conditions. The inlet shows the redox difference spectrum. (B) Cf-apo-cyt. b_6_ (3 μM) was mixed with heme (6 μM. (C) cf-cyt. b_6_-AB was mixed with cf-cyt. b_6_-CD in a one-to-one ratio (1 μM each), and heme (2 μM) was added in total protein to a heme ratio of one to one. (D) cf-cyt. b_6_-AB (2 μM) was mixed with heme (2 μM) and (E) cf-cyt. b_6_-CD (2 μM) was mixed with heme (2 μM). Note that different concentrations of protein and heme were used in (B) compared to (C), (D) and (E). The spectra in (B), (C) and (D) show the typical cyt. b_6_ absorbance maxima (ox: 562 nm / 531nm / 414 nm, red: 562 nm / 532 nm / 429 nm, redox: 562 nm / 532 nm for the α-/β-/γ-band).

To further support the assumption that the cyt. b_6_ BC-loop is dispensable for protein assembly, we next tested, whether the two cyt. b_6_ halves are able to bind the cofactors when the two halves are not covalently connected by a loop. Therefore, we aimed to express the two halves, AB and CD, independently. However, all attempts to heterologously express MBP-tagged fusion proteins in *E*. *coli* remained unsuccessful. Thus, the two cyt. b_6_ halves and, for the purpose of comparison, also the full-length cyt. b_6_, were recombinantly synthesized in a cell-free expression system. In the following, they are denoted “cf” (cell free)-cyt. b_6_-AB, cf-cyt. b_6_-CD and cf-cyt. b_6_, respectively. All three proteins were expressed without the MBP-fusion-tag, but with a deca-histidine-tag added at the respective N-terminus for purification. SDS-PAGE analyses of the samples before and after purification are shown in Fig 3A. Western blot analyses of the purified proteins are provided in the supplement (S2 Fig). After purification, both cf-cyt. b_6_ and cf-cyt. b_6_-AB ran as a distinct protein band in an SDS gel at about the expected molecular masses (26.9 kDa for cf-cyt. b_6_ and 15.5 kDa for cf-cyt. b_6_-AB) [70]. However, the purified cf-cyt. b_6_-CD protein ran as two distinct protein bands with apparent molecular masses of 14 and 15 kDa in about equimolar quantities. The same protein band pattern can be observed in the Western blot analysis when an anti-His-tag antibody was used, revealing that both proteins contain an intact N-terminal His-tag. However, with an antibody directed against the very C-terminus of cyt. b_6_, only the protein band at 15 kDa was detected in the Western blot analyses, and hence the 14 kDa protein band represents a C-terminally truncated version of cf-cyt. b_6_-CD, potentially generated due to premature termination of translation during *in vitro* protein synthesis. Initial attempts to separate these two peptides failed. However, as the C-terminal amino acids are unstructured and are not involved in protein assembly or heme binding [25, 27], we rationalized that the fragment, which misses some C-terminal amino acids, assembles and binds heme as the wt. This assumption was confirmed by the measurements presented and discussed below. Thus, the about equimolar mixture of the full-length and C-terminally truncated cf-cyt. b_6_-CD were used in the subsequent experiments.

**Fig 4:**
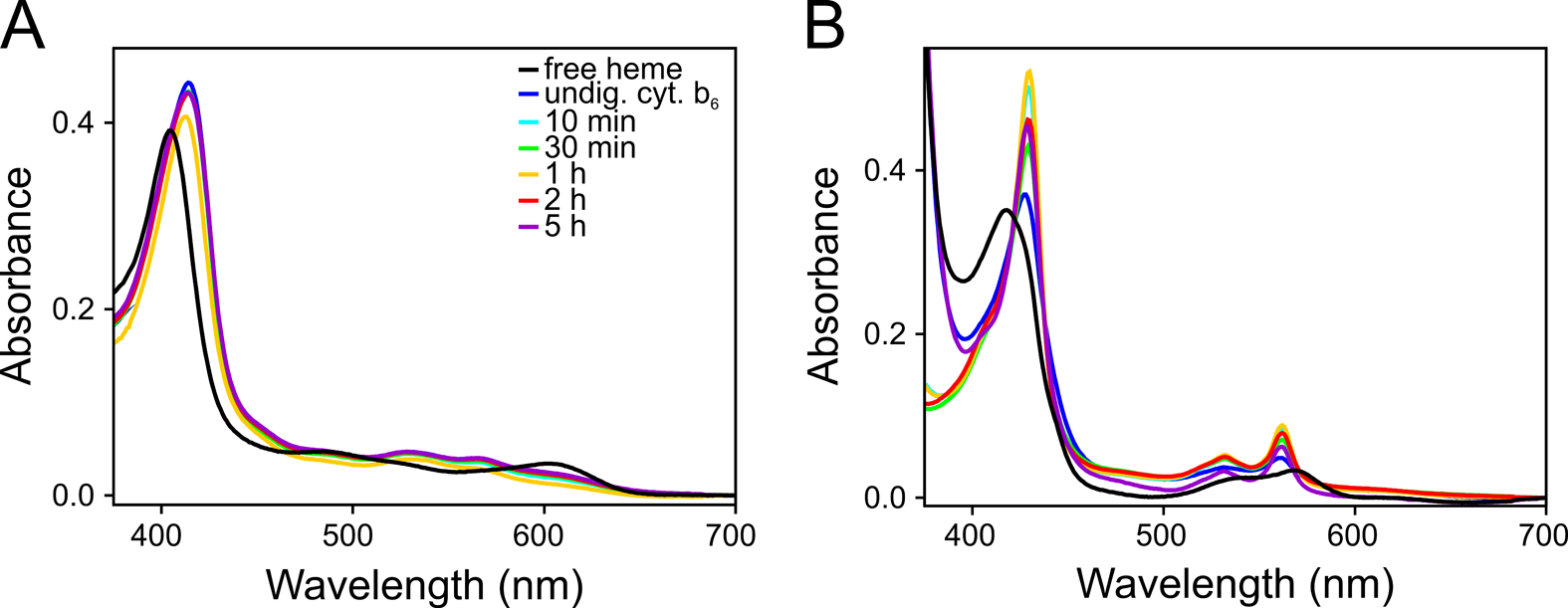
Absorbance spectra of cyt. b_6_ after proteolysis. (A, B) Holo-cyt. b_6_ was proteolytically digested with proteinase K. At defined time points (10 min light blue, 30 min green, 1 h yellow, 2 h red, 5 h purple) proteolysis was stopped and absorbance spectra were measured under oxidizing (A) and reducing conditions (B). As a control, the absorbance spectra of free heme (black) and reconstituted, undigested cyt. b_6_ (dark blue) were measured. The color legend in (A) also applies to (B). All spectra show the cyt. b_6_ characteristic maxima (ox: 562 nm / 531nm / 414 nm, red: 562 nm / 532 nm / 429 nm, for the α-/β-/γ-band).

To demonstrate that cf-apo-cyt. b_6_ can be refolded and reconstituted *in vitro* as properly as the MBP-fused protein, absorption spectra were measured after addition of heme to the purified cf-expressed protein (Fig 3B). Under both, oxidizing and reducing conditions, cf-holo-cyt. b_6_ displayed red-shifted and sharpened absorbance bands when compared to free heme (Fig 4A and 4B). These absorption characteristics were already detected before with the heterologously expressed cyt. b_6_-MBP protein (Fig 2A and 2B). Also the redox difference spectra resemble the spectra of the MBP-fused protein. The absorbance band maxima are red-shifted and sharpened compared to free heme (Fig 4A and 4B). This demonstrates that holo-cyt. b_6_ spontaneously assembles after addition of heme, and thus, the cell-free expressed cf-cyt. b_6_ protein behaves the same way as the fusion protein expressed in *E*. *coli*. Next, cyt. b_6_ was assembled from the two cf-cyt. b_6_ halves. The two halves were mixed in equal amounts and heme was added in a total-protein-to-heme-ratio of one to one (Fig 3C). Again, absorbance maxima characteristic for cyt. b_6_ were recorded under oxidizing and reducing conditions as well as a characteristic redox difference spectrum, and the bandwidth *fwhm* value decreased. Thus, heme is tightly bound by a cyt. b_6_-like protein assembled from the two cyt. b_6_ halves.

It is important to mention that the amount of cf-cyt. b_6_-CD that contributes to the total protein-to-heme-ratio in Fig 3C and 3E was calculated based on a cf-cyt. b_6_-CD concentration that includes both, the full-length as well as the C-terminally truncated CD fragment (Fig 3A). As we did not observe any characteristics of free heme in Fig 3C, such as a blue shift of the maxima, in the absorption spectra, C-terminal truncation of the cf-cyt. b_6_ CD did clearly not abolish heme binding.

In summary, the two cyt. b_6_ halves bind heme without being linked by a loop. Consequently, our data indicated that the BC-loop does not significantly influence cyt. b_6_ assembly and, therefore, is not essential for holo-protein assembly. However, as each of the two cyt. b_6_ halves contains two heme-ligating histidine residues, which are located on TM helix B and D, respectively, it was conceivable that already a homodimer of one of the two cyt. b_6_ halves is able to bind heme. To tackle this possibility, each cyt. b_6_ halve was mixed separately with heme. Cf-cyt. b_6_-AB shows typical, well-defined cyt. b_6_-like absorption characteristics under both, oxidizing and reducing conditions (Fig 3D). Thus, already cf-cyt b_6_-AB alone seems to be able to bind heme and to form a cyt b_6_-like four-helix bundle structure. In contrast, while the absorption spectrum of cf-cyt. b_6_-CD under oxidizing conditions shows a slightly shifted Soret-band absorbance maximum at 414 nm after heme addition (Fig 3E), under reducing conditions, no well-defined absorbance bands were visible in the absorbance spectrum and only a broad band at 426 nm was evident. This indicates an imperfect and (if at all) only very limited binding of heme to cyt. b_6_-CD, when the cyt. b_6_-AB halve is not present. Thus, cf-cyt b_6_-CD might be able to weakly bind heme under oxidizing, but does not bind heme under reducing conditions. However, these observations now raised the question, which of the cyt b_6_ halves actually contributed to the absorbance spectra shown in Fig 3C, where the two halves were mixed equimolarly. Was a well-defined spectrum observed due to exclusive formation of cyt b_6_-AB homodimers? While we cannot completely exclude that a small amount of cyt b_6_-AB homodimers had formed, the spectral characteristics strongly indicate formation of a cyt b_6_-like protein from the two halves. As cyt b_6_-CD does form a rather unstable heme-binding homodimer (if at all) under oxidizing conditions and does not form a cyt b_6_-like structure at all under reducing conditions (Fig 3E), formation of a cyt b_6_-like structure solely by the cyt. b_6_-AB fragment would have left half of the heme molecules free in solution. Thus, the absorbance spectrum of the sample would be far less defined with more spectral contributions from free heme. Since this clearly is not the case, the majority of the dimers contributing to the absorbance spectra in Fig 3C seem to be heterodimers of cf-cyt. b_6_-AB und cf-cyt. b_6_-CD.

In summary, the two cyt. b_6_ halves bind heme without being linked by the BC loop. The cyt. b_6_-AB halve by itself can bind heme efficiently, when the cyt. b_6_-CD halve is not present, albeit the heterodimer of the AB and CD fragments appears to be more stable. Consequently, our data indicate that the BC-loop does not significantly influence assembly of a cyt. b_6_(-like) protein and, therefore, is not essential for holo-protein assembly.

### Cyt. b_6_ assembles from TM fragments

Since the BC-loop appears to be of no particular importance for assembly of cyt. b_6_, we next asked the question whether the cyt. b_6_ TM fragments are able to bind heme without any connecting loop. Therefore, apo-cyt. b_6_ was first reconstituted with heme and the holo-protein was proteolytically digested by addition of proteinase K, which efficiently digests proteins [71]. However, complete digestion was not expected, since the cyt. b_6_ TM fragments were embedded in a “shielding” detergent micelle and potentially also the amino acids close to the TM region were still protected by the detergent micelle. Digestion of holo-cyt. b_6_ by proteinase K was stopped at distinct time points and absorbance spectra were measured to analyze whether heme was still bound (Fig 4A and 4B). Even 5 h after digestion, the spectra displayed absorbance maxima characteristic for properly assembled holo-cyt. b_6_, demonstrating that the heme cofactors remained bound after proteolysis. Surprisingly, under reducing conditions the absorbance heights of the band maxima of digested holo-cyt. b_6_ were increased compared to the undigested protein and the peaks appeared to be sharpened. This might indicate that soluble regions slightly hinder formation of the most stable b-type cytochrome in the wt protein, and the four TM helices of cyt. b_6_ potentially pack more closely when the soluble regions are removed. SDS-PAGE analysis of the digested cyt. b_6_ protein showed that already after 10 min no protein band corresponding to the mass of the full-length protein was detected anymore, and only protein fragments with molecular masses in the range of 8–15 kDa appeared (S3A Fig). Importantly, a sample incubated for the same time without proteinase K did not show any sign of degradation, emphasizing that proteolysis of cyt. b_6_ was strictly due to proteinase K. Western blot analysis, using specific antibodies directed against the cyt. b_6_ C- or N-terminus, respectively, did not detect any protein after 5 h of digestion (S3B and S3C Fig). This indicates that, although the soluble protein regions were proteolytically removed, the characteristic absorbance spectra originate from a cyt. b_6_-like protein assembled from fragments that are still competent to bind heme.

However, do the cyt. b_6_ TM helices still bind the hemes during/after proteolysis or are other fragments involved in (artificial) heme binding? The S4A and S4B Fig show absorbance spectra of a cyt. b_6_ variant recorded under oxidizing and reducing conditions. This cyt. b_6_ mutant does not bind heme due to mutation of two heme ligating His residues (cyt. b_6_ H86A and H187A) [8]. As described before, the apo-protein was proteolytically digested with proteinase K. At defined time points, proteolysis was stopped by addition of PMSF and 5 μM heme was added before acquiring absorbance spectra under oxidizing and reducing conditions. All spectra show solely maxima, characteristic for free, unbound heme. This indicates that not only the cyt. b_6_ H86A H187A full-length protein but also the proteolytic fragments of this cyt. b_6_ variant are unable to bind heme. It can therefore be concluded that the cyt. b-type absorbance spectra shown in Fig 4 are due to binding of heme to the cyt. b_6_ TM four-helix bundle.

**Fig 5:**
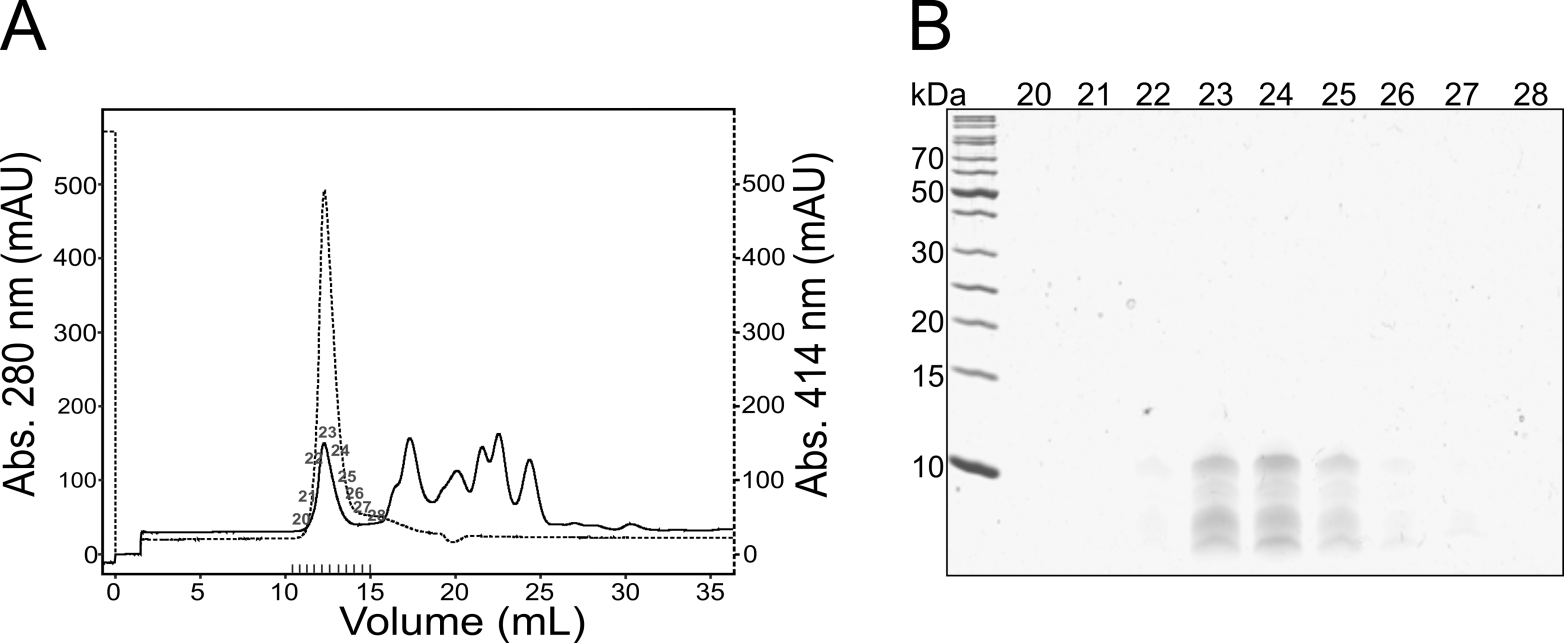
**Gel permeation chromatography (A) and SDS-PAGE-analysis (B) of proteolytically digested cyt. b**_**6**_. A gel permeation column was loaded with 0.5 mg cyt. b_6_ that was proteolytically digested with proteinase K for 5 h. Separation was done with 50 mM Tris pH 8, 50 mM NaCl and 1 mM DDM. The chromatogram (A) shows the absorption at 280 nm (continuous) and 414 nm (dashed) for protein and heme, respectively. Proteins in the elution fractions 20–28 (each 1 mL) were precipitated and the samples were separated on an 18% SDS gel (B).

To more clearly identify the cyt. b_6_ fragments involved in heme binding, reconstituted cyt. b_6_ was proteolytically digested with proteinase K for 5 h and the remaining protein fragments were separated by gel permeation chromatography. Absorbance was measured at 280 and 414 nm for detection of protein and heme, respectively (Fig 5A). At an elution volume of ~12.5 mL, a main peak appeared at both wavelengths, indicating that here protein fragments elute together with bound heme. Via SDS-PAGE-analyses we identified protein fragments with molecular masses in the range of 5–10 kDa (Fig 5B). The amino acid sequences of the fragments involved in heme binding after proteolysis were subsequently identified by mass spectrometry. A detailed list of all identified peptides can be found in the supplement (S2 Table). In this analysis, all four cyt. b_6_ TM helices were identified, albeit without the connecting interhelical AB- and CD-loops. The identified fragments correspond to TM helices A to D with some amino acids of the flanking loops remaining. Thus, proteinase K only digested the protein in the soluble regions, leaving the detergent-shielded TM helices intact. An exception of this is the TM helix B and the BC-loop. Some of the helix B fragments still include all the BC-loop amino acids and a few of the fragments even contained some amino acids of TM helix C. Thus, the detergent micelle seems to partly shield this loop from digestion since these amino acids likely do not protrude thus far into the bulk solution.

These results confirm the assumption that the cyt. b_6_ TM helices were largely protected against proteolysis by the detergent micelle. Although the helices were no longer connected via the interhelical AB- and CD-loops after proteolysis and only in part via the BC-loop, the heme cofactor remained bound and the cyt. b_6_ fragments were still able to properly bind the cofactors.

## Discussion

Cyt. b_6_ spontaneously binds two heme cofactors non-covalently, and *in vitro* assembly of the holo-protein can be followed spectroscopically [29, 30, 32]. On each helical hairpin of the heme-binding four-helix bundle (AB and CD), two His residues are conserved in one helix, and two of these His residues have predominant functions during heme binding [8]. Furthermore, Gly residues are conserved in the other TM helix of the helical TM hairpin, which are crucial for heme packing as they provide space for tight packing and an optimal fit for the heme [72]. The two helical hairpins (AB and CD) have an inverse TM topology, and thus, the di-heme cyt. b_6_ protein has an internal quasi-two-fold symmetry with the two related helical hairpins oriented at 180 degrees to each other [73].

A likely cyt. b_6_ folding pathway involves initial formation of the two helical-hairpins (helix pairs AB and CD) after integration of the TM helices into the membrane. Subsequently, the first heme (b_L_) binds [8]. Most likely, the structure of the four-helix bundle is not closed when the first heme (b_L_) is bound, since the second heme (b_H_) has to bind and the heme b_H_ binding niche has to be accessible. It appears to be likely that molecular interactions stabilize binding of heme b_L_ primarily at the surface of the helical hairpin formed by the TM helices A and B. Binding of the second heme (b_H_) is mediated and predominately stabilized by the heme-binding half-cavity, formed by the helical hairpin CD [8]. Binding of heme b_H_ obviously triggers formation of the final two heme-binding four TM helix bundle structure, which appears to be a prerequisite for subsequent assembly of the entire cytochrome b_6_f complex [48, 51]. Thus, formation of the two helical hairpin structures is crucial during assembly of the holo-protein. The two cyt. b_6_ halves have to be in close proximity to initially form an “open” heme binding cavity, which apparently tightens up after binding of the second b-type heme. Because of this, we initially anticipated that the loop region connecting the helical hairpins AB and CD might be vital for formation of a heme-accepting structure. The loop connecting the helical hairpins AB and CD mainly contains amino acids with relatively big side chains (Lys111, Lys112, Arg114 and Glu115), which limits the rotational freedom of the polypeptide chain. This results in a rather rigid conformation, which will distinctly position the two helical hairpins with respect to each other. The inflexible nature of the loop is further enhanced by the heterocyclic ring structure of proline (Pro113), which prevents free rotation of the C_α_-N-bond. However, as shown in the present study, elongation of the BC-loop had essentially no effect on the assembly and folding of cyt. b_6_, albeit the loop length was more than doubled. The small Gly side chains increased the flexibility of the BC-loop, and poly-Gly chains with a length of ten or more amino acids are known to have a freedom of movement comparable to a freely movable chain [74]. Thus, insertion of multiple Gly residues into the cyt. b_6_ BC-loop not only increased the distance between the cyt. b_6_ protein halves, but also severely enhanced the freedom of movement of the two helical hairpin structures. The cyt. b_6_-G_n_-mutants still bound two heme molecules with high affinity (Fig 2A, 2B and 2C). Hence, prolongation of the BC-loop does not severely affect the heme-binding equilibrium. Furthermore, when destabilized by successive addition of the harsh detergent SDS, all three proteins started releasing bound heme at ~4.5 mM SDS (Fig 2D), indicating a similar stability of the proteins. The close spatial proximity between the two cyt. b_6_ halves, which is induced by the relatively short loop, seems to not be crucial for high-affinity heme binding, and consequently the increased loop flexibility does not impair cofactor binding. These conclusions were further supported by the experiments with the separately produced cyt. b_6_ halves gained by cell-free expression. From these two individually structured halves, a stable holo-cyt. b_6_ structure can be assembled (Fig 3C). Thus, a covalent connection between the protein halves, correctly positioning the halves, is not essential for assembly. Furthermore, a cyt b_6_-like protein assembled even from solely the AB fragment, albeit formation of the ABCD structure appears to be favored. Together, TM helix-helix interactions and cofactor binding are sufficient for assembly of cyt. b_6_ from the two helical hairpins AB and CD, and the cyt. b_6_ BC-loop does not crucially affect protein assembly and stability.

But what about the remaining loops connecting TM helices AB and CD, respectively? To start defining the role of the covalent TM helix linkages on folding, assembly and stability of cyt. b_6_, we removed the soluble regions of holo-cyt. b_6_ by proteinase K (S3 Fig). The soluble regions of the full-length protein were largely proteolyzed after five hours, as judged by SDS-PAGE- and Western blot analyses. Nevertheless, the absorbance spectra did not significantly change (Fig 4A and 4B) and still indicated a six-fold coordination of the central heme iron, strongly suggesting that heme is still bound by the fragments, which remained after proteolysis. In fact, the spectra indicate that the heme environment is even better defined after proteolysis, and thus, the cyt. b_6_ structure appears to tighten up when soluble protein regions are cleaved off. The fragments responsible for the conserved heme-binding capacity were identified by mass spectrometry, and identified as the four cyt. b_6_ TM helices without the interhelical AB- and CD-loops. Solely some B helices carried an intact BC-loop after proteolysis. This is probably due to partial shielding of this short loop by the detergent micelle against the protease. Importantly, the before described experiments clearly exclude that this loop was crucial for formation of a b-type cytochrome structure. Taken all of these results into account, the TM helices seem to be able to stay assembled *in vitro* after cleavage of the interhelical loops and the cleaved fragments are able to bind heme. This indicates that assembly of cyt. b_6_ is mainly driven by interhelical interactions, like specific Van der Waals interactions, salt bridges or hydrogen bonds.

## Conclusion

Interhelical loops are not vital for assembly of the TM cyt. b_6_, and thus, the four-helix bundle is mainly stabilized by interhelical interactions in the TM region. In the past, the structural organization of the four TM helices of the cyt. b_6_ protein and of the homologous first four helices of cytochrome b have often served as a template for the design of water-soluble, di-heme-binding cytochrome models [42–44, 60, 75]. For enhancing van der Waals packing of the heme to the protein moiety, topologically defined four-helix bundles were designed and synthesized, which closely mimic the heme-binding core of cyt. b_6_, *i*.*e*. helices B/D and A/C, respectively [45]. Here, two different helices were synthesized: a heme-binding helix, which contains the conserved His and Arg residues involved in heme binding, as well as a second, heme-shielding helix, which contains the conserved Gly residues crucial for heme packing. However, the two helices were covalently linked, mimicking the helical hairpin structure found in cyt. b_6_. In contrast to a membrane environment, assembly of a water-soluble four-helix bundle is mainly driven by formation of the hydrophobic bundle core. Consequently, further optimization of heme-binding properties within the hydrophobic membrane environment is needed.

In recent years, heme-binding four-helix bundles have been designed that reside within the hydrophobic environment of micelles or lipid bilayers [40, 76, 77]. However, the designed proteins bound heme with a rather low affinity, indicating that the forces driving assembly of b-type cytochromes severely differ in a polar aqueous *vs*. non-polar membranous environment. As shown in the present study, specific TM helix-helix interactions likely result in formation of a TM cyt. b_6_ holo-protein structure, competent for high-affinity heme binding. Thus, the future design of heme-binding TM four-helix bundles having optimized heme-binding properties within the hydrophobic membrane environment should include the design of helix bundle structures with stable TM helix-helix interactions, which create a predefined heme-binding cavity.

## Supporting information

S1 Table Primer sequences. Sequences of the primers used for insertion of Gly residues into the BC loop. Gly-codons are in bold, restriction sites are underlined.
(DOCX)

S2 Table Cyt. b_6_ fragments identified by mass spectrometry after proteolytic digestion. All cyt. b_6_ fragments identified by mass spectrometry containing the sequence of a single cyt. b_6_ TM helix either in full-length or part of it with some amino acids of the flanking loops. An exception of this is TM helix B since some helix B fragments still contain the complete sequence of the BC-loop and a few of these fragments also contain amino acids of TM helix C. The TM areas are printed in bold. Amino acid residues flanking the detected peptides are indicated in parentheses.
(DOCX)

S1 Fig Expression and purification of cyt. b_6_ and the G_n_-mutants containing prolonged BC loops. Proteins were separated on 14% SDS gels. (A) cyt. b_6_, (B) cyt. b_6_-G_5_, (C) cyt. b_6_-G_10_. G_5_ and G_10_ stands for the number of Gly residues inserted into the BC loop. M: molecular mass standard; C: total cell extract before induction (control); CE: total cell extract before harvesting; S: soluble protein fraction; IB: inclusion body fraction; AC: protein purified by affinity chromatography.
(TIF)

S2 Fig Western blot analysis of purified cf-cyt. b_6_, cf-cyt. b_6_-AB and cf-cyt. b_6_-CD. Ni-NTA purified proteins were separated on an 18% SDS gel and blotted on a PVDF membrane. For immunologic detection, antibodies directed against the cyt. b_6_ N-terminus (A, B), cyt. b_6_ C-terminus (C) or His-tag (D) were used.
(TIF)

S3 Fig SDS-PAGE and Western-blot analysis of proteolytically digested holo-cyt. b_6_. Proteolysis of reconstituted holo-cyt. b_6_ (A, B, C) was stopped at various times. Proteins were separated on 18% (A, B, C) SDS gels and blotted on PVDF membranes (B, C). For immunological detection, antibodies directed against the cyt. b_6_ N-terminus (B) or cyt. b_6_ C-terminus (C) were used. PK: Proteinase K, C: cyt. b_6_ before digestion, NC: cyt. b_6_ incubated 5 h without proteinase K (negative control), 10: 10 min, 30: 30 min, 1h: 1 h, 2h: 2 h, 5h: 5 h of digestion.
(TIF)

S4 Fig Absorbance spectra of cyt. b_6_ H86A H187A after proteolysis. (A, B) Apo-cyt. b_6_ H86A H187A was proteolytically digested with proteinase K. At defined time points (10 min light blue, 30 min green, 1 h yellow, 2 h red, 5 h purple) proteolysis was stopped by addition of PMSF and 5 μM heme was added before acquiring absorbance spectra under oxidizing (C) and reducing conditions (D). As a control, the absorbance spectra of free heme (black) and reconstituted, undigested cyt. b_6_ (dark blue) were measured. All spectra of cyt. b_6_ (digested or not) show the maxima characteristic for free heme (ox: 603 nm / 405 nm, red: 566 nm / 541 nm / 418–420 nm, for the α-/β-/γ-band).
(TIF)
