# Supplementary material for: The soluble loop BC region guides, but not dictates, the assembly of the transmembrane cytochrome b6
Source: PLoS One. 2017 Dec 14;12(12):e0189532. doi: 10.1371/journal.pone.0189532 (PMC5730185; doi:10.1371/journal.pone.0189532)
Supplement: S1 Table — Sequences of the primers used for insertion of Gly residues into the BC loop. Gly-codons are in bold, restriction sites are underlined. (DOCX) [file pone.0189532.s001.docx]

| No. | Function | 5´ → 3´ Sequence |
| --- | --- | --- |
| 1 | Insertion of 5 G, fw | **GGAGGTGGAGGTGGA**CGAGAATTGACTTGGGTTACAGGC |
| 2 | Insertion of 5 G, rev | **TCCACCTCCACCTCC**AGGTTTTTTAAACCCACCGGTAAG |
| 3 | Insertion of 10 G, fw | **GGTGGAGGTGGAGGTGGAGGAGGTGGAGGT**CGAGAATTGACTTGGGTTACAGGC |
| 4 | Insertion of 10 G, rev | **ACCTCCACCTCCTCCACCTCCACCTCCACC**AGGTTTTTTAAACCCACCGGTAAG |
| 5 | N-terminus of *cyt b_6_* with SacI restriction site | GCAGACTAATTCGAGCTCGAACAAC |
| 6 | C-terminus of *cyt b_6_* with BamHI restriction site | CGCGGATCCCGTTGTAAAACGACGGC |
