# Supplementary material for: The soluble loop BC region guides, but not dictates, the assembly of the transmembrane cytochrome b6
Source: PLoS One. 2017 Dec 14;12(12):e0189532. doi: 10.1371/journal.pone.0189532 (PMC5730185; doi:10.1371/journal.pone.0189532)
Supplement: S2 Table — All cyt. b6 fragments identified by mass spectrometry containing the sequence of a single cyt. b6 TM helix either in full-length or part of it with some amino acids of the flanking loops. An exception of this is TM helix B since some helix B fragments still contain the complete sequence of the BC-loop and a few of these fragments also contain amino acids of TM helix C. The TM areas are printed in bold. Amino acid residues flanking the detected peptides are indicated in parentheses. (DOCX) [file pone.0189532.s002.docx]

| Length (AA) | MW (Da) | Start | End | TM helix | Sequence |
| --- | --- | --- | --- | --- | --- |
| 37 | 4267.27 | 434 | 471 | A | (S)KYVPPHVN**IFYALGGITLTAFLVQVATGF**AMTFYYRPT(V) |
| 41 | 4672.42 | 435 | 476 | A | (K)YVPPHVN**IFYALGGITLTAFLVQVATGF**AMTFYYRPTVTDAF(A) |
| 38 | 4339.29 | 435 | 473 | A | (K)YVPPHVN**IFYALGGITLTAFLVQVATGF**AMTFYYRPTVT(D) |
| 38 | 4355.27 | 435 | 473 | A | (K)YVPPHVN**IFYALGGITLTAFLVQVATGF**AMTFYYRPTVT(D) |
| 36 | 4155.16 | 435 | 471 | A | (K)YVPPHVN**IFYALGGITLTAFLVQVATGF**AMTFYYRPT(V) |
| 36 | 4139.17 | 435 | 471 | A | (K)YVPPHVN**IFYALGGITLTAFLVQVATGF**AMTFYYRPT(V) |
| 31 | 3458.83 | 435 | 466 | A | (K)YVPPHVN**IFYALGGITLTAFLVQVATGF**AMTF(Y) |
| 27 | 3072.60 | 444 | 471 | A | **(F)YALGGITLTAFLVQVATGF**AMTFYYRPT(V) |
| 27 | 3088.59 | 444 | 471 | A | **(F)YALGGITLTAFLVQVATGF**AMTFYYRPT(V) |
| 21 | 2498.28 | 450 | 471 | A | **(I)TLTAFLVQVATGF**AMTFYYRPT(V) |
| 42 | 5135.74 | 484 | 526 | B | (M)TEVN**FGWLIRSVHRWSASMMVLMMILHVFRVYLT**GGFKKPR**EL(T)** |
| 42 | 5151.74 | 484 | 526 | B | (M)TEVN**FGWLIRSVHRWSASMMVLMMILHVFRVYLT**GGFKKPR**EL(T)** |
| 33 | 4123.17 | 484 | 517 | B | (M)TEVN**FGWLIRSVHRWSASMMVLMMILHVFRVYLT**(G) |
| 31 | 3909.03 | 484 | 515 | B | (M)TEVN**FGWLIRSVHRWSASMMVLMMILHVFRVY**(L) |
| 41 | 5034.69 | 485 | 526 | B | (T)EVN**FGWLIRSVHRWSASMMVLMMILHVFRVYLT**GGFKKPR**EL(T)** |
| 41 | 5050.68 | 485 | 526 | B | (T)EVN**FGWLIRSVHRWSASMMVLMMILHVFRVYLT**GGFKKPR**EL(T)** |
| 36 | 4191.34 | 503 | 539 | BC | **(M)MVLMMILHVFRVYLT**GGFKKPR**ELTWVTGVVLGVLTA(S)** |
| 37 | 4222.32 | 506 | 543 | BC | **(L)MMILHVFRVYLT**GGFKKPR**ELTWVTGVVLGVLTASFGV(T)** |
| 17 | 2197.22 | 511 | 528 | B | **(H)VFRVYLT**GGFKKPR**ELTW(V)** |
| 42 | 4402.40 | 531 | 573 | C | **(T)GVVLGVLTASFGVTGY**SLPWDQIGYWAVKIVTGVPDAIPVIGS(P) |
| 36 | 4023.13 | 544 | 580 | C | **(V)TGY**SLPWDQIGYWAVKIVTGVPDAIPVIGSPLVELLR(G) |
| 34 | 3766.07 | 545 | 579 | C | **(T)GY**SLPWDQIGYWAVKIVTGVPDAIPVIGSPLVELL(R) |
| 30 | 3311.78 | 545 | 575 | C | **(T)GY**SLPWDQIGYWAVKIVTGVPDAIPVIGSPL(V) |
| 27 | 3014.62 | 545 | 572 | C | **(T)GY**SLPWDQIGYWAVKIVTGVPDAIPVIG(S) |
| 38 | 4395.42 | 575 | 613 | D | (P)LVELLRGSASVGQSTLTRFYS**LHTFVLPLLTAVFMLMHF(L)** |
| 40 | 4663.57 | 585 | 625 | D | (S)VGQSTLTRFYS**LHTFVLPLLTAVFMLMHFLMI**RKQGISGPL(-) |
| 39 | 4564.48 | 586 | 625 | D | (V)GQSTLTRFYS**LHTFVLPLLTAVFMLMHFLMI**RKQGISGPL(-) |
| 38 | 4507.48 | 587 | 625 | D | (G)QSTLTRFYS**LHTFVLPLLTAVFMLMHFLMI**RKQGISGPL(-) |
| 38 | 4523.47 | 587 | 625 | D | (G)QSTLTRFYS**LHTFVLPLLTAVFMLMHFLMI**RKQGISGPL(-) |
| 38 | 4523.47 | 587 | 625 | D | (G)QSTLTRFYS**LHTFVLPLLTAVFMLMHFLMI**RKQGISGPL(-) |
| 35 | 4256.30 | 587 | 622 | D | (G)QSTLTRFYS**LHTFVLPLLTAVFMLMHFLMI**RKQGIS(G) |
| 35 | 4240.32 | 587 | 622 | D | (G)QSTLTRFYS**LHTFVLPLLTAVFMLMHFLMI**RKQGIS(G) |
| 37 | 4379.42 | 588 | 625 | D | (Q)STLTRFYS**LHTFVLPLLTAVFMLMHFLMI**RKQGISGPL(-) |
| 37 | 4395.41 | 588 | 625 | D | (Q)STLTRFYS**LHTFVLPLLTAVFMLMHFLMI**RKQGISGPL(-) |
| 37 | 4411.39 | 588 | 625 | D | (Q)STLTRFYS**LHTFVLPLLTAVFMLMHFLMI**RKQGISGPL(-) |
| 34 | 4112.25 | 588 | 622 | D | (Q)STLTRFYS**LHTFVLPLLTAVFMLMHFLMI**RKQGIS(G) |
| 34 | 4144.24 | 588 | 622 | D | (Q)STLTRFYS**LHTFVLPLLTAVFMLMHFLMI**RKQGIS(G) |
| 31 | 3855.12 | 588 | 619 | D | (Q)STLTRFYS**LHTFVLPLLTAVFMLMHFLMI**RKQ(G) |
| 31 | 3871.12 | 588 | 619 | D | (Q)STLTRFYS**LHTFVLPLLTAVFMLMHFLMI**RKQ(G) |
| 29 | 3598.97 | 588 | 617 | D | (Q)STLTRFYS**LHTFVLPLLTAVFMLMHFLMI**R(K) |
| 36 | 4292.39 | 589 | 625 | D | (S)TLTRFYS**LHTFVLPLLTAVFMLMHFLMI**RKQGISGPL(-) |
| 36 | 4324.37 | 589 | 625 | D | (S)TLTRFYS**LHTFVLPLLTAVFMLMHFLMI**RKQGISGPL(-) |
| 36 | 4308.38 | 589 | 625 | D | (S)TLTRFYS**LHTFVLPLLTAVFMLMHFLMI**RKQGISGPL(-) |
| 33 | 4025.23 | 589 | 622 | D | (S)TLTRFYS**LHTFVLPLLTAVFMLMHFLMI**RKQGIS(G) |
| 33 | 4041.22 | 589 | 622 | D | (S)TLTRFYS**LHTFVLPLLTAVFMLMHFLMI**RKQGIS(G) |
| 30 | 3768.09 | 589 | 619 | D | (S)TLTRFYS**LHTFVLPLLTAVFMLMHFLMI**RKQ(G) |
| 28 | 3511.93 | 589 | 617 | D | (S)TLTRFYS**LHTFVLPLLTAVFMLMHFLMI**R(K) |
| 35 | 4223.33 | 590 | 625 | D | (T)LTRFYS**LHTFVLPLLTAVFMLMHFLMI**RKQGISGPL(-) |
| 35 | 4191.32 | 590 | 625 | D | (T)LTRFYS**LHTFVLPLLTAVFMLMHFLMI**RKQGISGPL(-) |
| 35 | 4207.33 | 590 | 625 | D | (T)LTRFYS**LHTFVLPLLTAVFMLMHFLMI**RKQGISGPL(-) |
| 32 | 3924.18 | 590 | 622 | D | (T)LTRFYS**LHTFVLPLLTAVFMLMHFLMI**RKQGIS(G) |
| 29 | 3667.03 | 590 | 619 | D | (T)LTRFYS**LHTFVLPLLTAVFMLMHFLMI**RKQ(G) |
| 29 | 3683.04 | 590 | 619 | D | (T)LTRFYS**LHTFVLPLLTAVFMLMHFLMI**RKQ(G) |
| 29 | 3683.03 | 590 | 619 | D | (T)LTRFYS**LHTFVLPLLTAVFMLMHFLMI**RKQ(G) |
| 27 | 3410.88 | 590 | 617 | D | (T)LTRFYS**LHTFVLPLLTAVFMLMHFLMI**R(K) |
| 27 | 3426.88 | 590 | 617 | D | (T)LTRFYS**LHTFVLPLLTAVFMLMHFLMI**R(K) |
| 24 | 3010.66 | 590 | 614 | D | (T)LTRFYS**LHTFVLPLLTAVFMLMHFL**(M) |
| 34 | 4078.26 | 591 | 625 | D | (L)TRFYS**LHTFVLPLLTAVFMLMHFLMI**RKQGISGPL(-) |
| 33 | 3977.21 | 592 | 625 | D | (T)RFYS**LHTFVLPLLTAVFMLMHFLMI**RKQGISGPL(-) |
| 30 | 3710.05 | 592 | 622 | D | (T)RFYS**LHTFVLPLLTAVFMLMHFLMI**RKQGIS(G) |
| 27 | 3452.91 | 592 | 619 | D | (T)RFYS**LHTFVLPLLTAVFMLMHFLMI**RKQ(G) |
| 32 | 3821.10 | 593 | 625 | D | (R)FYS**LHTFVLPLLTAVFMLMHFLMI**RKQGISGPL(-) |
| 26 | 3296.80 | 593 | 619 | D | (R)FYS**LHTFVLPLLTAVFMLMHFLMI**RKQ(G) |
| 31 | 3674.03 | 594 | 625 | D | (F)YS**LHTFVLPLLTAVFMLMHFLMI**RKQGISGPL(-) |
| 30 | 3510.97 | 595 | 625 | D | (Y)S**LHTFVLPLLTAVFMLMHFLMI**RKQGISGPL(-) |
| 30 | 3526.97 | 595 | 625 | D | (Y)S**LHTFVLPLLTAVFMLMHFLMI**RKQGISGPL(-) |
| 30 | 3526.97 | 595 | 625 | D | (Y)S**LHTFVLPLLTAVFMLMHFLMI**RKQGISGPL(-) |
| 24 | 2986.66 | 595 | 619 | D | (Y)S**LHTFVLPLLTAVFMLMHFLMI**RKQ(G) |
| 24 | 3002.67 | 595 | 619 | D | (Y)S**LHTFVLPLLTAVFMLMHFLMI**RKQ(G) |
| 24 | 3002.66 | 595 | 619 | D | (Y)S**LHTFVLPLLTAVFMLMHFLMI**RKQ(G) |
| 24 | 3002.67 | 595 | 619 | D | (Y)S**LHTFVLPLLTAVFMLMHFLMI**RKQ(G) |
| 22 | 2730.52 | 595 | 617 | D | (Y)S**LHTFVLPLLTAVFMLMHFLMI**R(K) |
| 28 | 3310.84 | 597 | 625 | D | **(L)HTFVLPLLTAVFMLMHFLMI**RKQGISGPL(-) |
| 28 | 3326.85 | 597 | 625 | D | **(L)HTFVLPLLTAVFMLMHFLMI**RKQGISGPL(-) |
| 28 | 3326.85 | 597 | 625 | D | **(L)HTFVLPLLTAVFMLMHFLMI**RKQGISGPL(-) |
| 28 | 3326.84 | 597 | 625 | D | **(L)HTFVLPLLTAVFMLMHFLMI**RKQGISGPL(-) |
| 25 | 3043.70 | 597 | 622 | D | **(L)HTFVLPLLTAVFMLMHFLMI**RKQGIS(G) |
| 22 | 2786.54 | 597 | 619 | D | **(L)HTFVLPLLTAVFMLMHFLMI**RKQ(G) |
| 22 | 2802.54 | 597 | 619 | D | **(L)HTFVLPLLTAVFMLMHFLMI**RKQ(G) |
| 22 | 2802.54 | 597 | 619 | D | **(L)HTFVLPLLTAVFMLMHFLMI**RKQ(G) |
| 27 | 3173.79 | 598 | 625 | D | **(H)TFVLPLLTAVFMLMHFLMI**RKQGISGPL(-) |
| 21 | 2649.49 | 598 | 619 | D | **(H)TFVLPLLTAVFMLMHFLMI**RKQ(G) |
| 25 | 2925.67 | 600 | 625 | D | **(F**)**VLPLLTAVFMLMHFLMI**RKQGISGPL(-) |
| 23 | 2713.52 | 602 | 625 | D | **(L)PLLTAVFMLMHFLMI**RKQGISGPL(-) |
| 17 | 2189.23 | 602 | 619 | D | **(L)PLLTAVFMLMHFLMI**RKQ(G) |
| 18 | 2236.22 | 604 | 622 | D | **(L)LTAVFMLMHFLMI**RKQGIS(G) |
| 20 | 2390.30 | 605 | 625 | D | **(L)TAVFMLMHFLMI**RKQGISGPL(-) |
| 17 | 2123.14 | 605 | 622 | D | **(L)TAVFMLMHFLMI**RKQGIS(G) |
| 14 | 1866.00 | 605 | 619 | D | **(L)TAVFMLMHFLMI**RKQ(G) |
| 16 | 2022.09 | 606 | 622 | D | **(T)AVFMLMHFLMI**RKQGIS(G) |
| 13 | 1764.96 | 606 | 619 | D | **(T)AVFMLMHFLMI**RKQ(G) |
| 18 | 2218.21 | 607 | 625 | D | **(A)VFMLMHFLMI**RKQGISGPL(-) |
| 11 | 1594.84 | 608 | 619 | D | **(V)FMLMHFLMIRKQ(G)** |
