# Supplementary figures and images for: The soluble loop BC region guides, but not dictates, the assembly of the transmembrane cytochrome b6
Source: PLoS One. 2017 Dec 14;12(12):e0189532. doi: 10.1371/journal.pone.0189532 (PMC5730185; doi:10.1371/journal.pone.0189532)

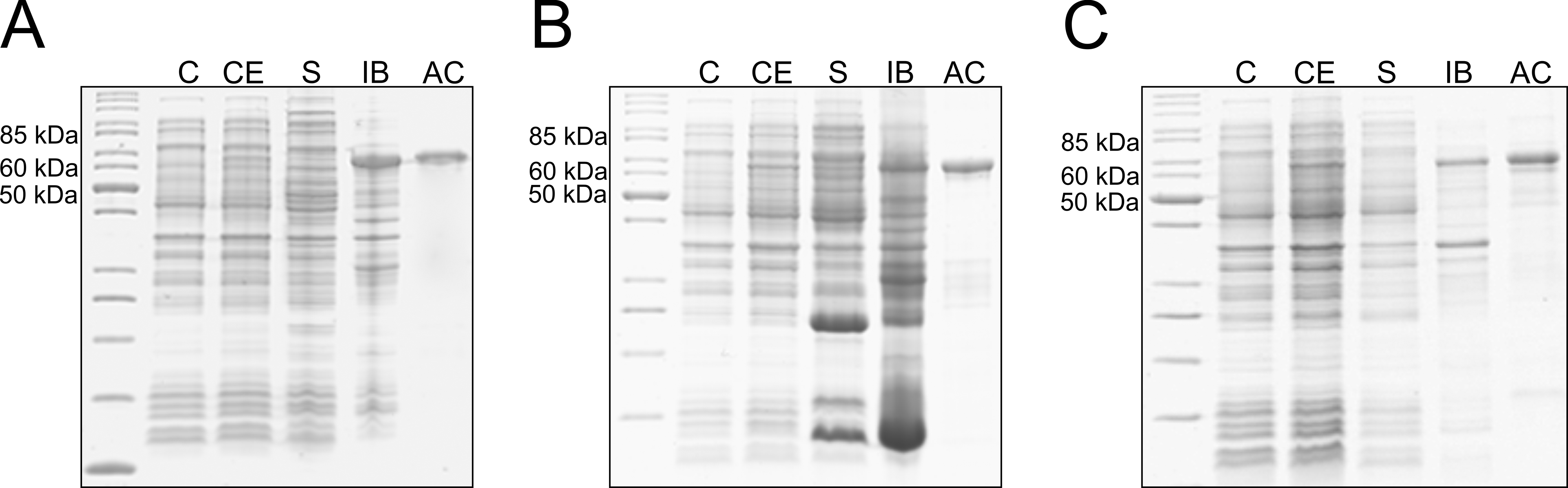

Supplement: S1 Fig — Proteins were separated on 14% SDS gels. (A) cyt. b6, (B) cyt. b6-G5, (C) cyt. b6-G10. G5 and G10 stands for the number of Gly residues inserted into the BC loop. M: molecular mass standard; C: total cell extract before induction (control); CE: total cell extract before harvesting; S: soluble protein fraction; IB: inclusion body fraction; AC: protein purified by affinity chromatography. (TIF) [file pone.0189532.s003.tif]

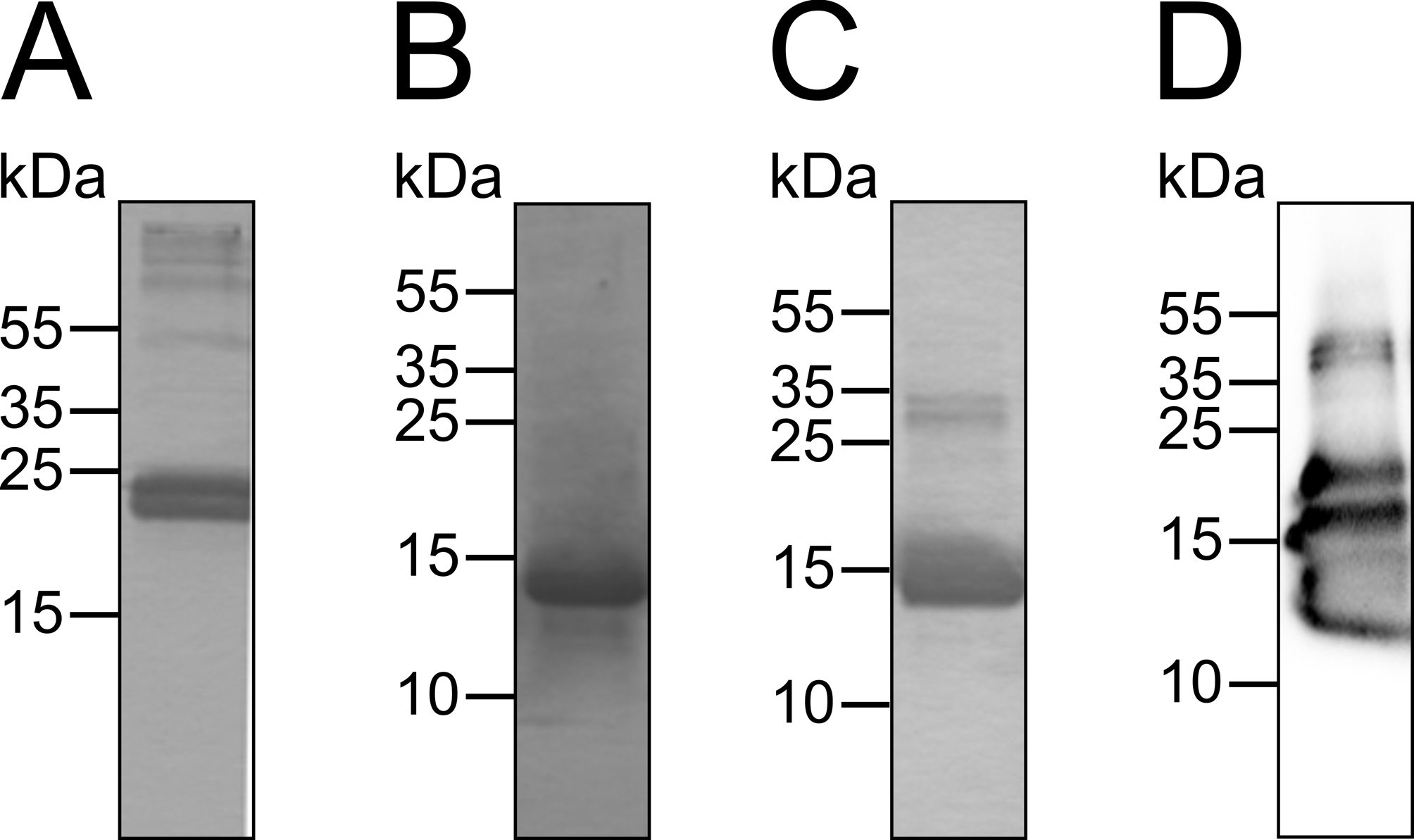

Supplement: S2 Fig — Ni-NTA purified proteins were separated on an 18% SDS gel and blotted on a PVDF membrane. For immunologic detection, antibodies directed against the cyt. b6 N-terminus (A, B), cyt. b6 C-terminus (C) or His-tag (D) were used. (TIF) [file pone.0189532.s004.tif]

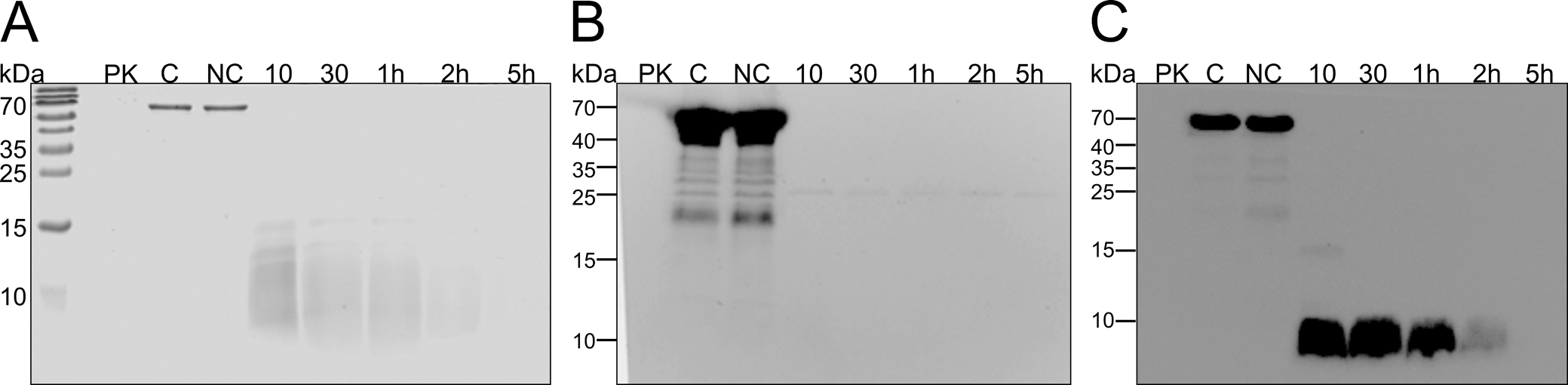

Supplement: S3 Fig — Proteolysis of reconstituted holo-cyt. b6 (A, B, C) was stopped at various times. Proteins were separated on 18% (A, B, C) SDS gels and blotted on PVDF membranes (B, C). For immunological detection, antibodies directed against the cyt. b6 N-terminus (B) or cyt. b6 C-terminus (C) were used. PK: Proteinase K, C: cyt. b6 before digestion, NC: cyt. b6 incubated 5 h without proteinase K (negative control), 10: 10 min, 30: 30 min, 1h: 1 h, 2h: 2 h, 5h: 5 h of digestion. (TIF) [file pone.0189532.s005.tif]

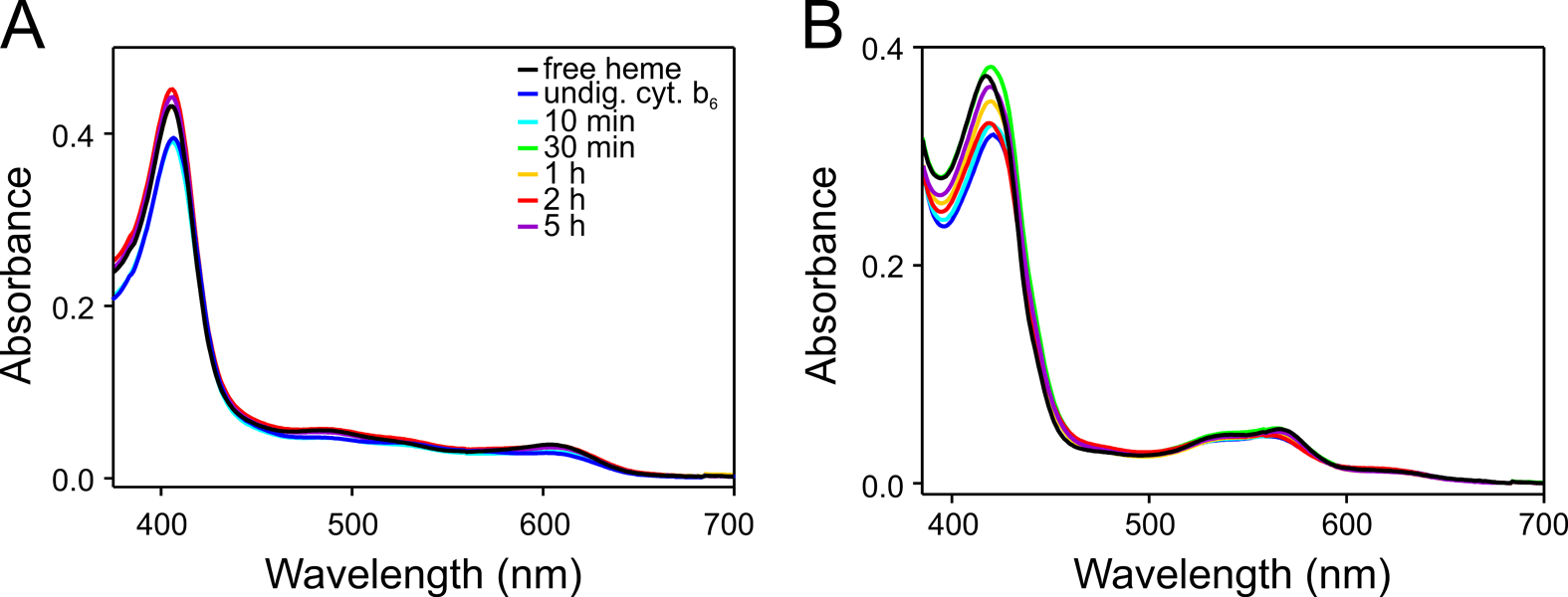

Supplement: S4 Fig — (A, B) Apo-cyt. b6 H86A H187A was proteolytically digested with proteinase K. At defined time points (10 min light blue, 30 min green, 1 h yellow, 2 h red, 5 h purple) proteolysis was stopped by addition of PMSF and 5 μM heme was added before acquiring absorbance spectra under oxidizing (C) and reducing conditions (D). As a control, the absorbance spectra of free heme (black) and reconstituted, undigested cyt. b6 (dark blue) were measured. All spectra of cyt. b6 (digested or not) show the maxima characteristic for free heme (ox: 603 nm / 405 nm, red: 566 nm / 541 nm / 418–420 nm, for the α-/β-/γ-band). (TIF) [file pone.0189532.s006.tif]
